# The distribution of robotic surgery in general and visceral surgery departments in Switzerland – a nationwide inquiry

**DOI:** 10.1515/iss-2023-0052

**Published:** 2024-02-19

**Authors:** Andreas Stalder, Federico Mazzola, Michel Adamina, René Fahrner

**Affiliations:** Department of Medicine, Hospital of Fribourg, Fribourg, Switzerland; Department of General and Transplant Surgery, University Hospital Zürich, Zürich, Switzerland; Department of Surgery, Hospital of Winterthur, Winterthur, Switzerland; Department of Vascular Surgery, University Hospital Bern, University of Bern, Bern, Switzerland

**Keywords:** robotic surgery, general surgery, visceral surgery, minimally invasive surgery

## Abstract

**Objectives:**

Since its introduction as a clinical technique, robotic surgery has been extended to different fields of surgery. However, the indications as well as the number of robotic procedures varied in different institutions. The aim of this investigation was to evaluate the current use of robotic surgery in general and digestive surgery in Switzerland.

**Methods:**

All Swiss surgical departments that are recognized training institutes for postgraduate training in surgery by the Swiss Institute of Medical Education (SIWF) were queried with a detailed questionnaire regarding the use of robotic surgery techniques and were analyzed regarding hospital size and type of hospital.

**Results:**

Ninety-three departments were queried, and 67 % (n=63) answered the survey. Fifty-eight were public, and five were private institutions. Seventeen (26 %) of the queried departments used robotic surgery in digestive surgery. Four out of 17 (23 %) of the departments that performed robotic surgery were private hospitals, while 13 (77 %) were public institutions. In the majority of departments, robotic surgery of the rectum (n=12; 70.6 %) and colon (n=11; 64.7 %) was performed, followed by hernia procedures (n=8; 47.1 %) and fundoplication (n=7; 41.2 %). Less frequently, pancreatic resections (n=5; 29.4 %), cholecystectomy (n=4; 23.5 %), adrenalectomy (n=4; 23.5 %), gastric bypass (n=3; 17.7 %), gastric sleeve (n=3; 17.7 %), hepatic procedures (n=2; 11.7 %), or small bowel resections (n=1; 5.9 %) were performed as robotic procedures. More than 25 procedures per year per department were performed for hernia surgery (n=5 departments), gastric bypass (n=2 departments), cholecystectomy, fundoplication, and colon surgery (each n=1 department).

**Conclusions:**

The number and range of robotic procedures performed in Switzerland varied widely. Higher accreditation for general surgery or subspecialization of visceral surgery of the department was positively associated with the use of robotic techniques, reflecting an unequal availability of robotic surgery.

## Introduction

The definition of robotic surgery varies among authors, but most authors honor Kwoh et al. as executing the first robotic procedure in 1988. Kwoh used a stereotactic robotic system to perform a CT-guided brain biopsy, a procedure that requires minimal hand movement [1]. In the 1980s, the US military recognized the potential significance of linking surgeons distant from the engagement area to surgical patients via a robotic platform. A collaboration between the Ames Research Centre at NASA and Stanford University led to the development of a robotic platform that was able to enhance and augment a laparoscopic procedure [2].

In 1997, the first robotic cholecystectomy was performed on a Da Vinci^®^ system in Belgium, which marked the beginning of robotic surgery in general and in visceral surgery in particular [3]. The first telerobotic procedure was carried out in 1999, whereby a cholecystectomy in Strasbourg was performed from a console in New York. This operation became known as the “Lindbergh operation” in reference to Lindbergh’s transatlantic flight [4].

Based on this research, two companies began to develop a commercially available robotic surgery system: Computer Motion with the ZEUS^®^ system and Intuitive Surgical with the Da Vinci^®^ system. In 2003, Intuitive Surgery acquired Computer Motion, and thus, only the development of the Da Vinci^®^ platform was continued. Henceforth, the Da Vinci^®^ system became the most widely used robotic laparoscopic system [5].

**Figure 1:**
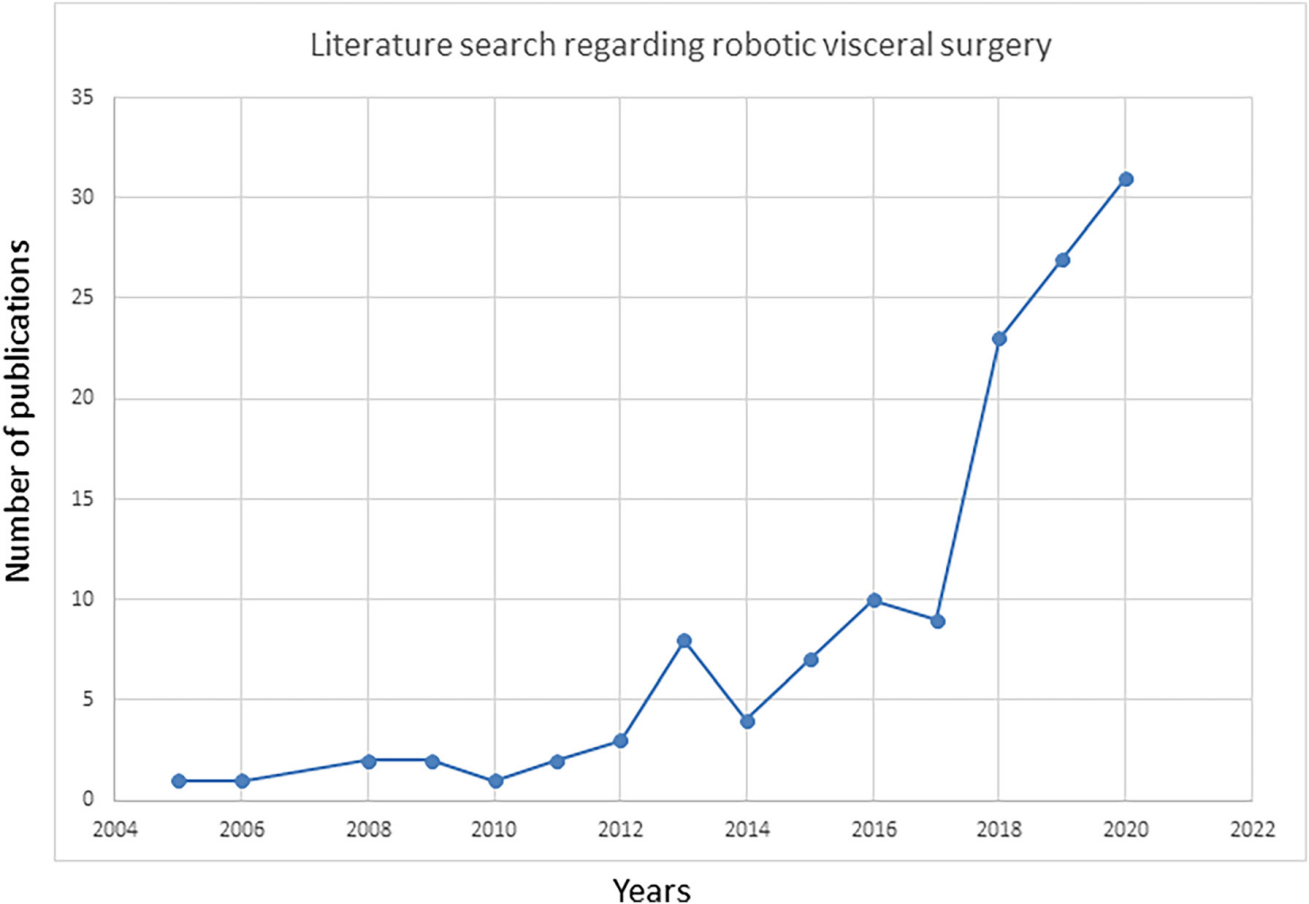
Pubmed results for the search query “robotic surgery” and “visceral” per year.

The first robotic surgery in Switzerland, a robotic prostatectomy, was performed in 2002 in Zurich by Prof. John Hubert [6]. While robotic surgery was initially predominantly used in urology, interest in robotic digestive surgery has grown over time, which can be attested to by the published number of publications in “PubMed” (Figure 1).

Since 2002, robotic surgery has spread all over Switzerland. In 2018, 33 “Da Vinci” systems were reported in Switzerland [7]. In recent years, robotic surgery has been expanded to several surgical fields and is also widely used in visceral surgery. However, the indications for the use of robotic surgery as well as the number of procedures in different institutions vary and are not publicly available. Therefore, the aim of this investigation was to register the current use of robotic systems in general and visceral surgery in Switzerland as a national centralized registry is missing so far and an overview of performed procedures around the different regions and hospitals is lacking.

## Materials and methods

From July to December 2020, we conducted a nationwide survey including all public and private hospitals listed in the registry of the Swiss Institute of Medical Education (SIWF), which is the accrediting body for medical specialties in Switzerland. All heads of department (n=93) were requested to fill out a questionnaire sent by post and email in the corresponding national language of each region (German, French, Italian). A reminder was sent after 8 and 12 weeks. Finally, departments that did not answer the second reminder were followed up by telephone.

The SIWF is responsible for all issues regarding training regulations and categorizes surgical departments based on their structure, size, and surgical expertise. Training centers for general surgery are divided into four categories: A and B1–B3. Additionally, training centers for the subspecialization in digestive surgery are subdivided according to their characteristics into V1–V3. Details are outlined in Table 1 [8, 9]. Furthermore, departments were categorized into public and private surgical departments depending on the ownership of the hospital, e.g., local or cantonal authority, university, or private owner.

**Table 1:** Categorization of surgical departments according to size, spectrum, and availability of related specialties accredited by the Swiss Institute of Medical Education (SIWF). Adapted according to the SIWF training reglementary for surgery.

Categorization of surgical departments [8, 9]		
Category level	Number of years of training credited to the training curriculum for general surgery	Description of the category level
B1	1	Surgical departments at smaller hospitals with a regular surgical activity, including an emergency operation
B2	2	Surgical clinics or departments at regional hospitals or corresponding institutions offering a wide range of surgical care around the clock
B3	3	Surgical clinics at cantonal hospitals, large regional hospitals, or corresponding institutions offering the entire surgical spectrum except for highly specialized areas
A	4	Large surgical clinics and departments at university and center hospitals with a broad spectrum and integrated interdisciplinary services

	Number of years of training credited to the subspecialty of visceral surgery	

V3	1	Small hospitals that offer a minimal spectrum of visceral surgery. They are led by a surgeon with the specialization of visceral surgery and have a 24 h/7 gastroenterology service as well as a standardized education program for the continuous formation
V2	2	V3 departments and additional own certified intensive care unit
V1	2	V2 departments and interventional radiology department available 24 h per day

### Questionnaire

The questionnaire surveyed the hospital size and accreditation level and listed 13 common digestive surgical procedures. The queried robotic procedures were appendectomy, cholecystectomy, fundoplication, inguinal hernia repair, thyroidectomy, small bowel resection, colonic resection, rectal resection, liver surgery, adrenalectomy, pancreatic resection, gastric bypass, and gastric sleeve. The listed procedures were not specified in further detail. We intentionally kept a broad definition, as we anticipated a considerable variety of approaches to each of the listed procedures among the different institutions.

### Ethics

Due to the character of this investigation with the collection of nonpersonal data via a questionnaire, ethical committee approval was waived. Data analysis was performed according to the guidelines of the Ethics Committee of Northwest and Central Switzerland.

### Statistical analyses

The data were collected and compiled in a descriptive manner. Categorical variables were reported as proportions and compared with the χ^2^ test. Statistical analysis of the data and graphics were performed with GraphPad Prism 5.0 software package (GraphPad, San Diego, California, USA), and a p ≤0.05 was assumed as statistically significant.

## Results

Overall, 63 departments answered the questionnaire, for a return rate of 67 %. There were differences in response rates by linguistic region, with 76 % of the German-, 50 % of the Italian-, and 33 % of the French-speaking departments answering the survey. The majority of departments were public institutions (n=58, 92 %). One quarter of the (n=16) surgery departments had the highest accreditation level A, and one fifth (n=13) had the highest subspecialization accreditation in visceral surgery V1. The characteristics of the participating departments are outlined in Table 2.

**Table 2:** Characteristics of departments that answered the questionnaire.

	Total	No robotic surgery		Robotic surgery		p-Value^a^
Institution characteristics						

Public	58	45	*78 %*	13	*22 %*	0.021
Private	5	1	*20 %*	4	*80 %*	

Accreditation level						

A	16	6	*38 %*	10	*62 %*	0.003
B3	14	11	*79 %*	3	*21 %*	
B2	24	21	*88 %*	3	*12 %*	
B1	9	8	*89 %*	1	*11 %*	

Subspecialisation accreditation						

V1	13	2	*15 %*	11	*85 %*	0.001
V2	14	10	*71 %*	4	*29 %*	
V3	4	3	*75 %*	1	*25 %*	
None	32	31	*97 %*	1	*3 %*	

Predominant language spoken in the department						

German	55	41	*75 %*	14	*25 %*	0.1
French	5	4	*80 %*	1	*20 %*	
Italian	3	1	*33 %*	2	*67 %*	

p-Value analyzed by χ^2^ test.

Seventeen (26 %) of the queried departments reported routine use of robotic surgery in general and visceral surgery. The first department started to use robotic surgery for general and visceral surgery procedures in 2014. Except for one, all departments performing robotic surgery were accredited for visceral surgery (V1 n=11, V2 n=4, V3 n=1). Similarly, most departments offering robotic surgery bared the highest accreditation in general surgery (n=10, 62 %; p=0.003), while a majority also had the highest accreditation in the subspecialty of visceral surgery (n=11, 85 %; p=0.001). The majority of surgical departments performing robotic surgery were located in public hospitals (n=13, 77 %), but almost all private surgical departments that answered the query used a robotic platform (n=4, 80 %; p=0.021, Table 2). Private surgical departments had lower accreditation levels in general surgery, but they all held the highest accreditation level for visceral surgery. Overall, a higher accreditation for general surgery or subspecialization of visceral surgery of the department was positively associated with the use of robotic techniques.

Most departments performed robotic surgery of the rectum (n=12; 70.6 %) and colon (n=11; 64.7 %), followed by hernia procedures (n=8; 47.1 %), fundoplication (n=7; 41.2 %), pancreatic resections (n=5; 29.4 %), cholecystectomy (n=4; 23.5 %), adrenalectomy (n=4; 23.5 %), bariatric procedures (bypass n=3; 17.7 %, gastric sleeve n=3; 17.7 %), hepatic procedures (n=2; 11.8 %), or small bowel resections (n=1; 5.9 %). Appendectomy or thyroidectomy was not performed. A single surgical department out of 17 did not specify the surgical procedures performed robotically. More than 25 robotic procedures per year were performed for hernia surgery (n=5 departments; 29.4 %), gastric bypass (n=2 departments; 11.8 %), cholecystectomy, fundoplication, and colon (each n=1 department; 5.9 %; see Table 3).

**Table 3:** Number of robotic procedures performed per year and per department.

Procedure	Number of procedures performed per year				Number of departments (%)
	1–5	6–10	>10	>25	
Appendectomy	0	0	0	0	0
Cholecystectomy	1	2		1	4 (23.5 %)
Fundoplication	1	3	2	1	7 (41.2 %)
Hernia	0	1	2	5	8 (47.1 %)
Thyroidectomy	0	0	0	0	0
Small bowel	0	1	0	0	1 (5.9 %)
Colon	2	3	5	1	11 (64.7 %)
Rectum	1	5	6	0	12 (70.6 %)
Liver	2	0	0	0	2 (11.8 %)
Adrenalectomy	1	3	0	0	4 (23.5 %)
Pancreas	2	2	1	0	5 (29.4 %)
Gastric bypass	0	1	0	2	3 (17.7 %)
Gastric sleeve	0	2	1	0	3 (17.7 %)

**Figure 2:**
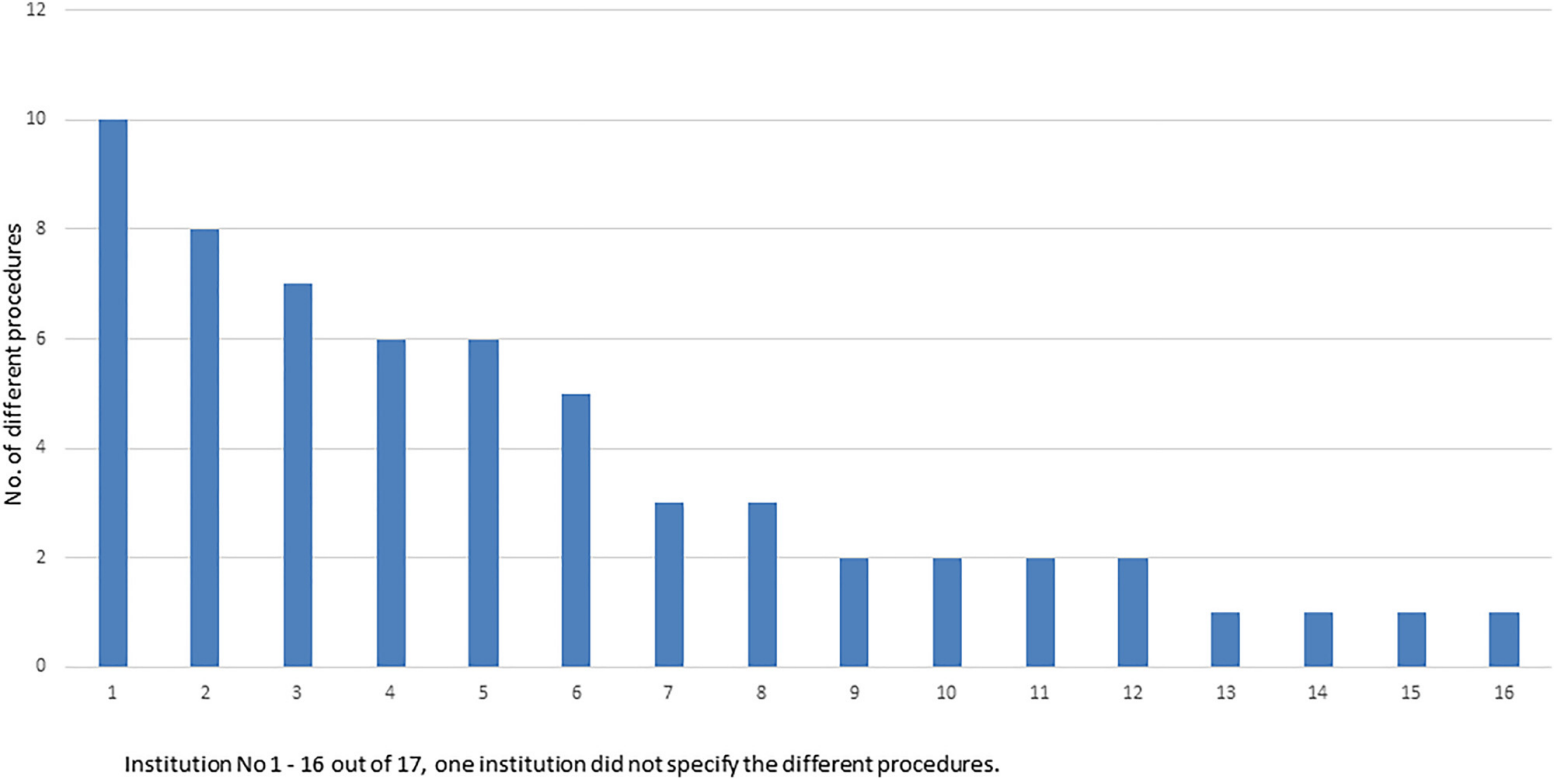
Spectrum of different procedures performed per surgical department.

Figure 2 illustrates the wide variation in the spectrum of robotic surgical procedures between the specific institutions. In six departments, more than five different procedures were performed, and three of these were private departments. One department stated that they had offered robotic visceral surgery in the past but stopped performing robotic surgery due to high costs.

## Discussion

This survey describes the current status of robotic surgery in general and visceral surgery in Switzerland. In a recently published nationwide survey in Switzerland regarding the distribution and access to laparoscopic surgery, the authors concluded that a strong disparity concerning demographic, geographic, and socio-economic issues restrict equity of access to innovative surgical care of the Swiss population [10]. While robotic surgery can be seen just as another evolution of minimally invasive approaches still lacking the proof of improved clinical outcomes, access to robotic platforms shall be equally available to all regions. In contrast to the current study of laparoscopic procedures, geographic differences concerning the three linguistic regions were not seen, but hospitals with higher accreditation for general and visceral surgery performed more frequently robotic procedures.

In this nationwide survey, approximately one-quarter of surgical departments reported the use of a robotic platform for general and digestive surgery. Especially for rectal, colon, and hernia procedures, robotic techniques were used. Most of the departments answered to use robotic platforms less than 25 times per year. The majority of surgical departments using robotic surgery were university hospitals or large cantonal hospitals, whereas smaller, regional hospitals did not perform robotic procedures. Interestingly, most of the private hospitals answering the query regularly used robotic surgery. It may be postulated that many robotic procedures are performed in the context of scientific studies or for plain marketing reasons. Furthermore, the wide range of interventions raises the assumption that robotic surgery is still being tested in many fields and not yet established for many procedures. Hence, a national registry recording all robotic procedures would provide a trusted overview of performed robotic approaches, results, and outcomes. Also, benefits and pitfalls of robotic approaches would become apparent through analysis of the registry.

Rectum and colon surgeries were performed with robotic techniques in most of the queried departments. Both robotic procedures have been shown to be safe in numerous studies regarding complication rates similar to those with a laparoscopic approach. Recent meta-analyses published in 2021 by Zhu et al. [11] and Kowalewski et al. [12] and in 2019 by Rausa et al. [13] suggested a reduction in the complication rate for robotic colorectal procedures compared to laparoscopic resections. However, such a reduction in morbidity was not confirmed in the largest randomized controlled study for colonic resection by Park et al. [14] in 2012 or in the only randomized controlled trial of rectal resection, the ROLARR-Study 2017 by Jayne et al. [15]. All authors highlighted the significantly higher costs of the robotic procedure in comparison to laparoscopic surgery [11], [12], [13], [14], [15]. Interestingly, only one department in the current study stated that they stopped robotic surgery due to higher costs. It may be postulated that many departments do not perform robotic surgery due to the high initial purchasing costs of a robotic platform. Unfortunately, due to the nature of this investigation, specific information regarding this issue is not available.

In the present investigation, robotic inguinal hernia surgery was the most often performed procedure in the participating departments. The most recent systematic review on robotic hernia surgery, which did not include any randomized controlled trial, was published in 2021 by Qabbani et al. This systematic review, which compared robotic inguinal hernia repair to open and laparoscopic inguinal hernia repair, suggested that the robotic technique was as safe and had fewer complications than the open and laparoscopic approaches. However, costs were significantly higher, and postoperative pain and recurrence rates were similar for robotic, laparoscopic, and open procedures [16]. Two randomized controlled trials, the PROVE-IT [17] and RIVAL [18] trials, showed similar results concerning clinical benefit and cost. Both noted no differences in key outcome parameters, such as complications, postoperative quality of life scores, and postoperative pain. In all mentioned studies, the considerably higher costs of the robotic procedure were outlined [16], [17], [18].

Interestingly, robotic fundoplication was performed in seven departments, and, in almost half of them, the procedure was performed more than 10 times per year and thus more frequently than cholecystectomies or bariatric surgery. A systematic review in 2021 by McKinley et al., including six randomized controlled trials, showed that robotic fundoplication was associated with higher costs yet failed to show significant differences in terms of outcome parameters [19]. Although bariatric surgery is on the rise, it is only performed in three departments as a robotic procedure. In the current literature, there is only one randomized controlled study comparing robotic and laparoscopic gastric bypass thus far [20]. A systematic review in 2018 by Wang et al. did not find significant differences regarding complication rates between the robotic and laparoscopic procedures [21]. For sleeve gastrectomy, there are no randomized controlled studies, yet a systematic review in 2017 by Magouliotis et al. found comparable outcomes for the robotic and laparoscopic procedures, whereas the duration of operation and the length of hospital stay were significantly longer in the robotic group than in the laparoscopic group [22].

In Switzerland, only five departments performed robotic pancreatic surgery; however, there was no differentiation in this investigation between pancreatic head and pancreatic tail resections. A systematic review comparing open vs. robotic pancreaticoduodenectomy showed a shorter length of hospital stay after robotic surgery but no differences in surgical complications [23]. A meta-analysis comparing open vs. robotic distal pancreatectomy showed a higher spleen preservation rate, lower conversion rate, and shorter hospital stay for the robotic technique [24]. In line with other studies investigating robotic surgery, robotic pancreatectomies were significantly more costly than laparoscopic or open approaches [23, 24].

Cholecystectomy was the very first procedure ever performed with a laparoscopic robotic system [4]. There is ample evidence about the safety of this procedure. Two systematic reviews, including a total of five randomized controlled trials published in 2018 by Han et al. and 2021 by Muaddi et al., showed no significant differences in intraoperative complications, postoperative complications, readmission rate, hospital stay, estimated blood loss, or conversion rates. In contrast, the duration of operation was significantly longer using robotic surgery in comparison to laparoscopic surgery [25, 26]. Interestingly, robotic cholecystectomy was least frequently performed in this survey. One reason may be that laparoscopic cholecystectomy is often performed as an entry teaching procedure in general surgery and, therefore, not offered as a robotic procedure [27, 28]. Additionally, Kadakia et al. postulated that a shift toward robotic procedures performed by senior residents or attending surgeons leads to a delay in residency training [29]. Another recent investigation revealed that young residents are interested in robotic surgery and would appreciate an early implementation of robotic surgery during residency [30]. A study from the United States showed that in many surgical residency programs, a robotic curriculum was already introduced in recent years [31]. Although the teaching aspect is an important issue why robotic surgery is not performed in many Swiss surgical departments, the higher costs and the apparent lack of advantages of this technique are probably additional reasons why many departments do not use robotic techniques.

Overall, cholecystectomies, hernia repair, and fundoplications were very frequently performed robotically. Potential reasons for this are that these operations are common and thus widely available in large numbers while also being highly standardized. Hence, these entry robotic procedures allow for a safe training of robotic surgeons prior to embarking to more complex procedures.

Robotic adrenalectomy and robotic liver surgery were only performed in four and two departments, respectively. In the current literature, it has been shown that robotic procedures for adrenalectomy and liver resection are safe and may have advantages regarding decreased length of hospital stay, reduced blood loss, fewer complications, and lower readmission rates [32], [33], [34], [35]. In the case of robotic liver surgery, robotic techniques may overcome the limitations of conventional laparoscopic liver surgery, mainly regarding the posterior–superior segments, due to improvements regarding articulation of the instruments during robotic procedures in comparison to rigid laparoscopic instruments [36].

Only one department reported using robotic techniques for small bowel resection, and no department performed appendectomies or thyroidectomies with robotic surgery. This fact is also reflected in the current literature, as no comparative studies have been published thus far regarding small bowel resections or appendectomy because of acute inflammation. There are reports of robotic appendectomy as part of other surgical procedures [37] and for appendiceal mucocele [38]. One reason not to use robotic surgery for appendectomy may be that these operations are often performed as a nonelective surgery at night or on weekends, with limited access to robotic platforms.

Robotic thyroidectomy has been described with different approaches and techniques [39, 40]. Therefore, a comparison to open or conventional minimally invasive techniques is difficult. In a systematic review comparing open and robotic approaches, complication rates were similar between both techniques, but robotic surgery was associated with reduced blood loss, a smaller number of retrieved lymph nodes, a lower level of swallowing impairment, and better cosmetic satisfaction. In contrast, the open procedure was associated with a shorter operation time, smaller total drain amount, and lower postoperative serum thyroglobulin levels [41].

A strength of the present study is its representativeness for its nationwide high response rate of 67 %. Indeed, most survey in the medical literature and even more in social science report return rate well below 50 % [42, 43], whereas response rate higher than 60 % are seen as highly representative of the queried population [44]. However, this study did not investigate the reasons why some institutions have adopted a robotic approach or, conversely, why other institutions refrained from offering robotic surgery. This survey only described the current nationwide status and performance of robotic surgery. A reliable estimation of the dynamic rate and possible changes in the use of robotic surgery cannot be drawn. Additionally, surgical outcomes were not assessed and a centralized registry of all robotic procedures comparable to other countries (e.g., Germany) would be welcome.

In conclusion, based on the reports of all responding departments, approximately one-quarter of Swiss surgical departments offer robotic general and digestive surgery. The number and range of robotic procedures offered differed significantly between institutions. SIWF-accredited private hospitals offered a broader spectrum of digestive robotic surgery than public hospitals. In addition, larger cantonal and university hospitals performed a broader spectrum and greater numbers of robotic procedures, which is reflected in the broader surgical spectrum and expertise in surgical procedures as well as scientific activities. So far, outcome analyses of robotic procedures from Switzerland are missing, so that comparable to the neighbor country Germany, a centralized registry (StuDoQ Robotik) of all performed robotic procedures in general and visceral surgery would be very welcome. In the future, the demand for robotic surgery will most likely increase, and access to robotic platforms is already available and will further increase as competition between institutions is increasing. Long-term registry and clinical trials of robotic surgery are likely to confirm benefits for a robotic approach for most if not all digestive surgical procedures. Hence, ultimately it can be expected that access to robotic platforms will become readily available in most hospitals and all regions of the country.
